# Homœopathy Fairly Represented

**Published:** 1854-05

**Authors:** 


ART. VII.—*Homœopathy fairly represented. A reply to Prof. Simpson's "Homœopathy" misrepresented.* By Wm. Henderson, M. D., Professor of General Pathology in the University of Edinburgh. Philadelphia: Lindsay & Blakiston. 1854.

So much space has recently been given to homœopathy in our columns, that we were somewhat averse to making any lengthy notice of Dr. Henderson's work. But the consideration of the source from whence it came, the most eminent and learned within the pale of homœopathy, disposed us to give the book a careful perusal, in hopes to find an honest and truthful exposition of homœopathy.

With this disposition we sat down to its perusal filled with the determination to read it through. We have not done so. We defy any man of common sense to go through it in course. If we could discover in it any attempt to elucidate the author's doctrines, we could endure any manner of style; but there is nothing, to us, so insufferably tedious as to wade through a volume of diatribe and malignant personality. If we wish to abuse any one we usually find a single page sufficient for our purpose; at any rate, we have a notion that disinterested readers generally tire at any larger amount.

As is evident from the review a correspondent furnished to our last number of Dr. Simpson's work, it is not in any sense a personal attack on Dr. Henderson, but a calm and honest statement of the differences between the two systems of medication. But Dr. Henderson chooses to make it an individual matter, to apply to Dr. Simpson sundry hard names, and to conduct himself like a very ill-tempered and angry man.

Dr. Henderson has very foolishly lost a golden opportunity. Dr. Simpson conferred dignity on the heresy by the pains with which he investigated it, and the good-tempered argument he maintained. Had Dr. Henderson done the same, had he gone into a calm and gentlemanly discussion of the real question, he would have gained an important advantage, for he would then have earned a right to be considered Dr. Simpson's equal; that gentleman having consented to meet him.

But sound reasoning, and quiet truth, are not the weapons of homœopathy; and accordingly we have only to record that if any one wishes to read three hundred pages of invective, got up on the model of our recent congressional quarrels, he can do so by investing a small sum in this book at Miller, Orton & Mulligans.

